# Human resources for research: building bridges through the Diaspora

**DOI:** 10.3402/gha.v8.29559

**Published:** 2015-11-06

**Authors:** Karen Hofman, Beverley Kramer

**Affiliations:** 1 School of Public Health, Faculty of Health Sciences, University of the Witwatersrand, Johannesburg, South Africa; 2 Health Sciences Research Office, Faculty of Health Sciences, University of the Witwatersrand, Johannesburg, South Africa

**Keywords:** research, human resources, Diaspora

## Abstract

**Background:**

The collaboration of scientists between the developed and the developing world is an opportunity to reverse the ‘brain drain' and to enable ‘brain circulation'.

**Objective:**

Attracting alumni from the Diaspora to strengthen the development of talented scientists will strengthen research in Africa.

**Design:**

In 2010, the Faculty of Health Sciences at the University of the Witwatersrand, South Africa (SA), created an Alumni Diaspora Programme to boost international research collaboration and networking between leading medical and health sciences alumni who now live and work at academic institutions abroad with academic colleagues ‘back home'. Based in Johannesburg, a gateway city attracting researchers from all over sub-Saharan Africa, this programme has the potential to capitalise on some of the intellectual capacity that was lost, mostly during the decades of apartheid, and to strengthen capacity, not just in SA, but across the continent.

**Results and Conclusions:**

The goal of this review is to highlight how this programme has stimulated collaborations and networking with international alumni.

The post-2015 era presents two important overarching challenges for science in the developing world – the first is to expand and apply our rapidly growing scientific knowledge for societal benefit and the second is to confront increasing global disparities in health and quality of life. The scientific research enterprise is at the convergence of these important global agendas but significant challenges exist. One of the enabling factors behind the transformation of human health in the past 100 years has been the expansion of available interventions as a result of research. The success of this endeavour relies in part on the creation of more opportunities for scientist-to-scientist interactions and strengthening institutional capacity. One of the key challenges is to enhance the sub-Saharan African (SSA) scientific research enterprise, bolster centres of excellence, and to encourage the development of a cadre of investigators able to advance a research agenda for the region.

## Research capacity in the SSA

The ‘10/90 Report on Health Research', which documents disparities between resources in rich and poor nations, suggests that strengthening research capacity is one of the most effective and sustainable ways of advancing health and development (1). Of 53 countries in the African continent, South Africa (SA) spends <1% of gross domestic product on medical research. Little of this is spent on capacity development (2). Although the South African Medical Research Council and the National Research Foundation have made efforts to enhance funding for research, the funding for the training of clinician scientists, in particular, remains limited.

Establishment of robust research institutions is critical for alleviating current disease burdens and taking advantage of the multitude of scientific opportunities. Fostering the evolution of local institutions into centres of excellence will help drive stronger, more sustainable science in the region.

Preparing SA investigators for such success requires strengthening capacity and expertise, along with the entire research cascade – from writing grant proposals and scientific manuscripts to research training and grant administration. Harnessing the untapped expertise of Diaspora scientists, who currently live and work abroad, would be one opportunity to strengthen capacity and increase research output. Researchers at universities in the United States agree that there is a need to build stronger and more sustained partnerships with African institutions with the recognition that such collaborations face real challenges. Some partnerships already occur as a result of the efforts of African Diaspora scientists working in major academic US institutions, who, on an *ad hoc* basis, facilitate international partnerships in their countries of origin. However, more sustainable coordination is needed and our Diaspora scientists should be looked upon as ‘recoverable assets' who can play an important role in developing skills and capacity in SA (3, 4). Collaborating with our alumni is one way of ‘thinking locally and acting globally' (5) and may inspire global action in solving local health problems through health research. The mobility of scientists or ‘brain circulation' may spur scientific progress globally (6). This review of the University of the Witwatersrand (Wits) Alumni Diaspora Programme shows how the model was created, demonstrates some of its benefits, and describes its challenges.

### The brain drain

Since the early 1990s, Africa has been losing 20,000 professionals each year, according to the International Organization for Migration. This trend resulted in recognition by the United Nations that ‘emigration of African professionals to the West is one of the greatest obstacles to Africa's development' (7). The beneficial contribution of the ‘one-way movement' of highly skilled academics from developing to developed countries has been acknowledged (8). Central to the problem of academic emigration are issues of supply, demand, and mobility, as well as limitation in specific areas of postgraduate education and career development, and comparatively poor working conditions for those scientists emanating from southern Africa (8). More specifically, these issues include large differences in remuneration and living conditions for work in the low-income countries, juxtaposed with a demand for skilled workers in the high-income countries. Political repression and instability are further reasons for emigration (9). In Canada alone, 297 African-born academics were employed in 124 institutions in 2008, whereas between 20,000 and 25,000 African-born academics were employed at academic institutions in the United States (10). International mobility could be developed to the benefit of both the importers of scientists as well as the exporters (6).

In SA, public health services have been weakened by the loss of human resources because of the accelerating ‘brain drain' abroad, as well as internally to the private sector. The loss of return on investment to SA has been estimated at approximately US$1.44 billion (accounting for the cost of education from primary school through completion of their basic medical studies, but not including specialist training) (11).

At Wits the brain drain has had an impact on health research. Since the first medical students were accepted in 1919, Wits has trained more than 10,000 medical doctors. In addition, the university has produced substantial numbers of practitioners in dentistry, nursing, pharmacy, public health, and occupational and physiotherapy. A substantial number of these individuals have gone on to perform basic clinical- and policy-relevant research. The faculty annually awards an average of nearly 400 undergraduate degrees and diplomas, and currently has approximately 500 medical specialists in training *per* annum. A significant number of these graduates have emigrated. Over two decades, from 1970 to 1990, approximately 3,600 medical doctors graduated from Wits (personal communication, Wits Alumni Office). Thirty per cent of Wits health sciences alumni left the country over a similar period (between 1975 and 1995), of which approximately half went to the United States. Currently, approximately 350 alumni (not all from one class) leave every year. The majority of doctors graduating from Wits have moved largely to four countries: the United States, Canada, Australia, and the United Kingdom. Many of these health professionals have pursued stellar careers in academia and have attained senior positions. Of 65 Wits Medical School paediatric graduates (1940–1979) who had immigrated to the United States, 31 achieved full professorships and 25 were chairpersons of academic departments and/or divisions (12). Wits health sciences alumni have established themselves as influential figures across the globe.

African academic institutions face challenges in executing key research functions. Wits has produced excellent researchers and continues to do so, but there is always a need to expand and improve. There are few pure research posts funded by academic institutions, which are largely public universities, in SA. Although all clinicians face the triple threat of administration, teaching, and research, the majority of SA clinicians experience a quadruple threat because of exceptionally high service demands. Less than 1% of clinicians at our institution have an MD, PhD though there has been a call in SA to increase the numbers of clinician scientists in particular (13). Some academics who have left their country of origin maintain relationships with academics ‘back home'. These individuals are able not only to play an important role in building new international partnerships but they also have a more nuanced understanding of all the issues, whether political, cultural, or linguistic, which may arise in their country of origin (14).

Although the developed world has greater access to resources which enable cutting-edge research, there are many opportunities for new research in the developing world. It is thus important for developing countries to participate in new (e.g. Ebola) and ongoing research (e.g. burgeoning lifestyle diseases and cancers) that are occurring in our continent (15). There are also major opportunities for alumni from the developed world to participate in public health research which is not only important to the developing world but has an impact on populations globally. Although Parthasarathi (16) holds that reverse flows of skilled scientific personnel are far smaller than emigration, recently Wiesel (17) reported that 70% of Fellows who participated in the Pew Latin American Fellows Program returned to their home countries despite lack of adequate resources for cutting-edge research. There are many examples of existing programmes for strengthening research ‘back home' from around the world, for example, China (18–21), India (18, 22), Latin America (23), Nigeria (14), and South Africa (24, 25). As Foulds and Zeleza (10) report, many Diaspora academics wish to share their skills with institutions on their home continent and see their relationship as a national service. The Wits Diaspora Programme described here not only allows for alumni to return ‘home' to participate in strengthening research in their home country but also allows for talented researchers at Wits to travel to a resource-rich setting to acquire and develop skills.

## Wits Alumni Diaspora Programme

Over a 4-year period (2010–2013) during the pilot stage with internal funding, we brought 22 alumni from the United States, Canada, Europe, and New Zealand back to Wits for short periods of time. In 2014, an application to fund the programme was awarded by the Carnegie Corporation of New York. As a major incentive in the new Wits Diaspora Programme, in partnership with the Carnegie Corporation of New York, Wits has been enabled to send local hosts on return visits to their Fellows abroad.

The Wits Alumni Diaspora pilot project has demonstrated concrete gains to Wits and to our alumni. International alumni have been delighted to re-engage with their alma mater with growing interest and many have commented on the opportunity to ‘give back'. There has been growing interest in the programme from international alumni. Several ongoing collaborations have been established, with alumni mentoring or supervising junior staff and postgraduate students. One of the most successful and fruitful engagements has been with an alumnus based at Vanderbilt University School of Medicine. This has resulted in an agreement at institutional level, the provision of intensive training on writing international grant applications and scientific papers, several collaborative research projects, grant applications, and substantial support for the establishment of a biomedical informatics initiative. Two further strong collaborations were established with The John Hopkins School of Medicine and the University of Queensland. In addition, there have been meaningful intangible gains for postgraduate students and emergent researchers through liaisons with international giants who have emanated from their alma mater. Academic and personal relationships have been forged and research collaborations have been initiated.

Although the programme is still young, we believe that the gains have been substantial.

In 2014–2015, our new association with the Carnegie Corporation of New York has allowed us to collaborate with a further 13 international alumni. Of a total of 35 returning alumni since the inception of the programme in 2010, 68% have been from the United States and Canada, 15% from the United Kingdom and Europe, and a further 14% from Australia and New Zealand. Collaborating with our alumni is one way of ‘thinking locally and acting globally' (6) and may inspire global action in solving local health problems through health research. The mobility of scientists or ‘brain circulation' may be an incentive for scientific progress globally (6).

The Alumni Diaspora Programme is beneficial both to Wits and to our international visitors, especially where longer term collaborations are initiated. One visiting alumnus from Canada said, for example, that ‘some of the most provocative questions' that she had encountered in her career originated at the talks she delivered at Wits. Another from Vanderbilt remarked that ‘there are areas where Wits is a world leader in research in both science and medicine'.

A key future focus will be to better align expectations of the host and alumnus/a's (sending) institution to maximise the bilateral gains. In addition to the alumnus/a, the sending institution should be made aware of the opportunities that are possible in SA for research. Policies need to be put in place at the sending institution to facilitate and enable tenured faculty members to devote time to such programmes. Resources for the sustainability of this programme are especially important for development of both staff and students, as this will not only have an impact on Wits but on the entire continent. Wits attracts students, particularly at the postgraduate level, from all over Africa, and their research training is of the utmost importance in developing health research capacity. Thus, our ‘returning' alumni have an important role to play in strengthening health research not only in SA but also throughout the African continent.

Although there were already some good international research collaborations at Wits prior to initiating this programme, there have been some specific concrete gains. This includes expanded contacts and collaborations at international institutions. A ripple effect has occurred with the SA alumni making further connections for our researchers with faculty at their institutions. This broader networking will hopefully support continued global health research into the future. As a result of these contacts, several joint grant applications have been submitted in 2015.

The programme has stimulated global connections/collaborations with a spin-off for global health. Identification of health issues which were thought only to be of relevance to SA has been broadened into joint global health research projects, thus elevating these collaborations into potential global solutions for global health.

There have been positive experiences for young faculty who have made return visits to Diaspora Fellows. For some, these visits have exposed them to first world infrastructure and skills which has in turn expanded their research vistas. One important lesson is that that the exposure abroad is relevant to local circumstances. For example, one young researcher found that they were unable to initiate the specific laboratory techniques learned with their Diaspora host, due to lack of infrastructure and funding at Wits. This type of exposure is, however, still of value.

The benefits of this programme have not been unidirectional. Some Diaspora Fellows have, during their visits, been exposed to Wits methods of postgraduate (master's level) protocol development and assessment. Several Fellows have expressed their admiration for these methods and have elected to pursue introduction of these formats at their institutions. However, in relation to capacity development, some advice received regarding training of PhD candidates, while interesting, is not always context specific. For example, a suggestion to extend the training time, variety of courses, and skills for PhDs is not feasible, especially for local clinicians, due to the burden of disease and the heavy service loads of our ‘clinician scientists' in training. Wits has implemented a training programme for clinician scientists which is addressing these issues (26).

As with any experimental programme, there have been, and will continue to be challenges. On the one hand, Wits hosts have not always followed through by pursuing their visiting alumnus/a. On the other hand, some visitors have not always engaged following visits. Some in the Wits community have also voiced concerns that the alumni are out of touch with their issues, with a changed SA, and with the university.

Over the past two decades since the end of apartheid, Wits has been in the forefront in SA of transforming the institution from both a sex and race perspective. Such evolution is essential in the advancement of higher education in the country. The Carnegie-Wits Alumni Diaspora Programme provides an additional scaffold for transformation by providing opportunities for networking and collaboration for faculty and postgraduate students.

## Conclusion

As an institution that emphasises ‘health research for health', a major challenge is the onerous service delivery expected of clinical staff that affects the time they have to carry out research. With the enormous range of patients and the wide diversity of diseases, this setting could benefit greatly from additional human resources in the form of skilled researchers participating in a multitude of projects while simultaneously contributing to the generation of scientific knowledge. The Wits Diaspora Programme has opened the possibility to build on existing networks. This has the potential for strengthening research capacities and health research not only among the health sciences faculty but also among our Diaspora alumni to mitigate the health problems of SA, of the continent, and of Diaspora communities across the globe.

### Relevance to other countries

The significant willingness from alumni abroad to participate and ‘give back' to their home institution has been astounding. This engaging attitude has resulted in the opening up of opportunities which may otherwise not have occurred. These opportunities through engagement with alumni are not specific to Wits or SA, but could be applied to all global institutions. The opportunities which have occurred through our programme relate to training opportunities, research collaborations, submission of joint grant applications, and intellectual input into local research projects resulting in expanded research design. In addition, exposure of local young researchers to high-quality international researchers and the institutions where many of them have made their mark is a form of ‘training' and experience which cannot be provided through any didactic course.

An international debate on the possibilities and opportunities for engaging the Diaspora in the SSA health sector could provide an explosion of relevant health research and transform ways of thinking in both directions.

## References

[1] Nchinda TC (2000). Chapter 7: capacity development for health research. The 10/90 Report on Health Research 2000.

[2] ICSU Regional Office for Africa (2007). Health and human well-being in sub-Saharan Africa.

[3] Kitua AY, Corrah T, Herbst K, Nyirenda T, Agwale S, Makanga M (2009). Strengthening capacity, collaboration and quality of clinical research in Africa: EDCTP networks of excellence. Tanzan J Health Res.

[4] Dodani S, LaPorte RE (2005). Brain drain from developing countries: how can brain drain be converted into wisdom gain?. J R Soc Med.

[5] Tarantola D (2013). Thinking locally, acting globally. Am J Pub Health.

[6] Hunter P (2013). Brain drain, brain gain or brain sharing?. EMBO Rep.

[7] Tebeje A (2005). Brain drain and capacity building in Africa.

[8] Oberoi SS, Lin V (2006). Brain drain of doctors from southern Africa: brain gain for Australia. Aust Health Rev.

[9] Foulds K, Zeleza PT (2014). The African academic Diaspora and African higher education. Int Higher Educ.

[10] Foulds K, Zeleza P (2015). Harnessing the potential of Africa's Global Academic Diaspora.

[11] Mills E, Kanters S, Hagopian A, Bansback N, Nachega J, Alberton M (2011). The financial cost of doctors emigrating from sub-Saharan Africa: human capital analysis. BMJ.

[12] Gilchrist G (2004). In the footsteps of Abraham Jacobi, an early international medical graduate: contributions of a single South African medical school to US Pediatrics. Pediatrics.

[13] Mayosi BM, Dhai A, Folb P, Gevers G, Hussey G, Kirkman M (2009). Revitalising clinical research in South Africa: a study on clinical research and related training.

[14] Anand NP, Hofman KJ, Glass RI (2009). The globalization of health research: harnessing the scientific Diaspora. Acad Med.

[15] Ng M, Fleming T, Robinson M, Thomson B, Graetz N, Margono C (2014). Global, regional, and national prevalence of overweight and obesity in children and adults during 1980–2013: a systematic analysis for the Global Burden of Disease Study 2013. Lancet.

[16] Parthasarathi A (2006). Turning brain drain into brain circulation. IAEA Bull.

[17] Wiesel T (2014). Fellowships: turning brain drain into brain circulation. Nature.

[18] Seguin B, Singer PA, Daar AS (2006). Scientific Diaspora. Science.

[19] Xin H (2006). Scientific workforce. Frustrations mount over China's high-priced hunt for trophy professors. Science.

[20] Cha AE (2008). Opportunities in China lure scientists home. The Washington Post.

[21] Zweig D Is China a magnate for talent? (PowerPoint document).

[22] Morton CC Public health mandate is broad, says Cutter Lecturer K. Srinath Reddy. Harvard Public Health Now.

[23] Chaparro F, Jaramillo H, Qunitero V (2009). Role of Diaspora in facilitating participation in global knowledge networks: lessons of Red Caldas in Colombia.

[24] Cherry M (2007). South African scheme lures in top talent. Nature.

[25] Pretorius C Funding for science research diverted. Mail & Guardian Online.

[26] Kramer B, Veriava Y, Pettifor J (2015). Rising to the challenge: training the next generation of clinician scientists for Africa. Afr J Health Prof Educ.

